# Characterization and morphological methods for oral biofilm visualization: where are we nowadays?

**DOI:** 10.3934/microbiol.2024020

**Published:** 2024-06-06

**Authors:** Davide Gerardi, Sara Bernardi, Angelo Bruni, Giovanni Falisi, Gianluca Botticelli

**Affiliations:** 1 Department of Life, Health and Environmental Sciences, University of L'Aquila, L'Aquila, Italy; 2 Department of Biotechnological and Applied Clinical Sciences, University of L'Aquila, L'Aquila, Italy

**Keywords:** microbial detection, microscopy techniques, oral biofilm, oral microorganism

## Abstract

The oral microbiome represents an essential component of the oral ecosystem whose symbiotic relationship contributes to health maintenance. The biofilm represents a state of living of microorganisms surrounding themselves with a complex and tridimensional organized polymeric support and defense matrix. The substrates where the oral biofilm adhere can suffer from damages due to the microbial community metabolisms. Therefore, microbial biofilm represents the main etiological factor of the two pathologies of dental interest with the highest incidence, such as carious pathology and periodontal pathology. The study, analysis, and understanding of the characteristics of the biofilm, starting from the macroscopic structure up to the microscopic architecture, appear essential. This review examined the morphological methods used through the years to identify species, adhesion mechanisms that contribute to biofilm formation and stability, and how the action of microbicidal molecules is effective against pathological biofilm. Microscopy is the primary technique for the morphological characterization of biofilm. Light microscopy, which includes the stereomicroscope and confocal laser microscopy (CLSM), allows the visualization of microbial communities in their natural state, providing valuable information on the spatial arrangement of different microorganisms within the biofilm and revealing microbial diversity in the biofilm matrix. The stereomicroscope provides a three-dimensional view of the sample, allowing detailed observation of the structure, thickness, morphology, and distribution of the various species in the biofilm while CLSM provides information on its three-dimensional architecture, microbial composition, and dynamic development. Electron microscopy, scanning (SEM) or transmission (TEM), allows the high-resolution investigation of the architecture of the biofilm, analyzing the bacterial population, the extracellular polymeric matrix (EPS), and the mechanisms of the physical and chemical forces that contribute to the adhesion of the biofilm to the substrates, on a nanometric scale. More advanced microscopic methodologies, such as scanning transmission electron microscopy (STEM), high-resolution transmission electron microscopy (HR-TEM), and correlative microscopy, have enabled the evaluation of antibacterial treatments, due to the potential to reveal the efficacy of different molecules in breaking down the biofilm. In conclusion, evidence based on scientific literature shows that established microscopic methods represent the most common tools used to characterize biofilm and its morphology in oral microbiology. Further protocols and studies on the application of advanced microscopic techniques are needed to obtain precise details on the microbiological and pathological aspects of oral biofilm.

## 1. Introduction

Oral biofilm is a complex and dynamic microbial community that forms on the surfaces of teeth and other oral structures, identified as niches, thus the tongue, the subgingival niche, the dental prosthesis, and the niche around dental implants [1].

**Figure 1.**
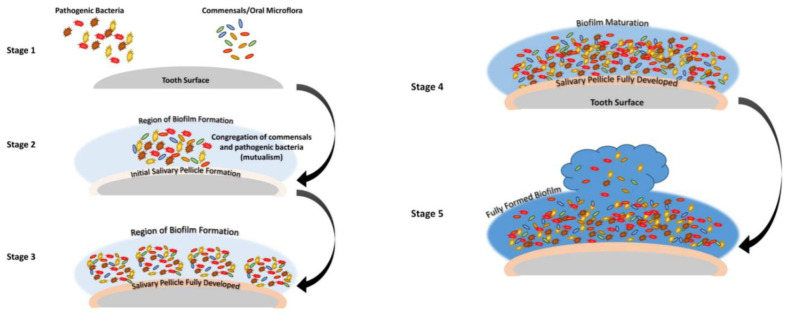
The stages of oral biofilm. Stages 1–2: The adhesion phase lasts approximately 3–5 hours, which involves an initial reversible adsorption of the bacterial cells to the substrate and a subsequent attachment via the acquired salivary film. Stages 3–4: The maturation phase is completed in vitro in approximately 12–48 hours, which goes from the formation of the first microcolonies to the completion of the mature biofilm, with the formation of macro-colonies. These are the result of the growth of bacterial cells and the production of the extracellular matrix by the same cells. Dysbiosis is completed. Stage 5: The dispersion phase involves the detachment of single cells or groups of cells that can colonize surrounding or distant sites and promote the origin of a new biofilm [4].

The process of oral biofilm formation begins with the attachment of early colonizers, such as *Streptococcus species*, to the tooth surfaces. The chemical and physical characteristics of the substrates play an essential role in the formation of oral biofilm, which is mediated by the different forces of adhesion, such as Van der Waals forces, ionic interactions, quorum sensing, Brownian motion, surface tension, adhesion, and cohesion. These initial adhesions pave the way for establishing a diverse microbial ecosystem. As the biofilm matures, other bacteria, including *Actinomyces* and *Fusobacterium species*, join the community. Therefore, microorganisms exist in a structured and organized manner, with distinct layers of bacteria based on their metabolic activities. While some bacteria thrive in oxygen-rich environments near the tooth surface, others, such as anaerobic species like *Porphyromonas gingivalis*, prefer deeper layers where oxygen is limited [2]. The matrix created by the extracellular polymeric substances (EPS) is the oral biofilm's main characteristic and protects the microbial community [3] (Figure 1).

The health of the oral cavity as an ecosystem depends on the equilibrium between the microorganisms and the structures of the oral cavity. Thus, the oral biofilm is a normal and essential component of the oral ecosystem. It aids in maintaining oral health by preventing the colonization of harmful bacteria and promoting a balanced microbial community. However, if not adequately controlled through oral hygiene practices, the biofilm can become pathogenic, leading to dental diseases such as caries and periodontal disease. In the presence of different oral pathologies, oral biofilm changes the types of microorganism species and their organization [5].

Oral biofilm, which primarily constitutes bacteria, EPS, and salivary proteins, results from the interactions of different chemical, physical, and biological components within the framework. Bacteria, both gram-positive and gram-negative, form the bulk of the biofilm; *Streptococcus mutans*, *Porphyromonas gingivalis*, and *Actinomyces species* are among the numerous microbial species in oral biofilm. These bacteria adhere to the tooth surfaces and each other through various mechanisms, creating a diverse and organized community.

EPS is pivotal in the structural integrity of oral biofilm, forming a matrix composed of polysaccharides, proteins, and nucleic acids that encases and protects bacterial cells. This matrix also aids in adhesion to tooth surfaces, facilitating the establishment and persistence of the biofilm, and represents a dynamic scaffold that supports the three-dimensional architecture of oral biofilm [6].

Salivary proteins, such as mucins and enzymes, contribute to the chemical composition of oral biofilm. Mucins provide a lubricating and protective layer on tooth surfaces, fostering initial bacterial adhesion, while enzymes in saliva participate in the breakdown of dietary substances, influencing the metabolic activities of bacteria within the biofilm. Additionally, antimicrobial proteins in saliva play a role in the host's defense against pathogenic bacteria in the oral cavity [7].

The formation and stability of oral biofilm involves a combination of various chemical and physical forces. The chemical forces that contribute to the adhesion of bacteria to surfaces, the maintenance of the biofilm structure, and the interactions between microbial cells are Van der Waals forces, hydrogen bonding, ionic interactions, and quorum sensing (Table 1) and the physical forces are Brownian motion, surface tension, adhesion, and cohesion (Table 2).

**Table 1.** A summarizing table of the chemical forces and related descriptions of their actions and roles in biofilm formation.

Chemical Forces	Description of their actions and roles in biofilm formation
Van der Waals forces	Weak attractive forces between molecules, including those present on bacterial surfaces, playing a key role in the initial adhesion of bacteria to tooth surfaces [8].
Hydrogen bonds	Forces between molecules with hydrogen and electronegative atoms. Regarding the oral biofilm, hydrogen bonding can occur between water molecules, proteins, and other biomolecules, influencing the structure and stability of the biofilm [8].
Ionic interactions	Electrostatic interactions between charged molecules or groups which can affect bacterial adhesion. The charged molecules on bacterial and tooth surfaces can lead to attractive or repulsive forces [9].
Quorum sensing	A chemical communication system used by bacteria to coordinate gene expression based on population density. Quorum sensing allows bacteria within the biofilm to respond collectively to environmental changes and regulate biofilm formation [10].

**Table 2.** A summarizing table of the physical forces and related descriptions of their actions and roles in biofilm formation.

Physical Forces	Description of their actions and roles in biofilm formation
Brownian motion	The random motion of particles, such as bacteria, due to thermal energy contributing to bacteria transport within the oral environment and colliding with tooth surfaces [11].
Surface tension	The tendency of the surface of a liquid to resist external forces. In the oral environment, the surface tension of saliva can influence the spreading and wetting of oral biofilm on tooth surfaces [12].
Adhesion and cohesion forces	Forces contributing to the structural integrity of the biofilm. Adhesion forces refer to the attachment of bacteria to tooth surfaces or other structures, while the cohesion ones involve bacteria binding to each other within the biofilm [13].

The bacterial composition of the oral microbiome is primarily dominated by six phyla: *Firmicutes*, *Bacteroidetes*, *Proteobacteria*, *Actinobacteria*, *Spirochaetes*, and *Fusobacteria*, representing oral cavity bacteria. Other significant constituents of the oral microbiome include *Actinobacteria* (genera *Actinomyces*, *Atopobium*, *Corynebacterium*, and *Rothia*), *Bacteroidetes* (*Bergeyella*, *Capnocytophaga*, and *Prevotella*), *Firmicutes* (*Streptococcus*, *Granulicatella*, and *Veilonella*), *Proteobacteria* (*Campylobacter*, *Cardiobacterium*, *Haemophilus*, *Aggregatibacter*, and *Neisseria*), and *Fusobacteria*. The initial colonizers of the oral cavity belong to the genera *Streptococci* (e.g., *S. mitis* and *S. salivarius*) and *Fusobacterium* [14],[15].

These early colonizers play a significant role in the proliferation of other species and the formation of the biofilm facilitated by the products of their metabolism.

The oral biofilm is classified, in relation to the main niches, into 5 types: supragingival, plaque on the tongue, subgingival plaque, plaque on the dental prosthesis, and plaque around the implant (Table 3).

**Table 3.** The composition of oral bacterial biofilm supragingival, plaque on the tongue, and subgingival [16],[17].

Supragingival	Plaque on the tongue	Subgingival
· *Actinomyes*	· *Prevotella*	- The outside layer: Treponemes
· *Aggregatibacter*	· *Rothia*	
· *Fusobacterium*	· *Neisseria*	- The first layer (on the top of the biofilm):
· *Leptotrichia*	· *Veillonella*	· *Actinomyces species*
· *Capnocytophaga*	· *Porphyromonas*	· Bacteria belonging to the *Cytophaga-Flavobacterium-Bacteroides* cluster
· *Corynebacterium*	· *Granulicatella*	
· *Lautropia*	· *Alloprevotella*	- The intermediate layer:
· *Campylobacter Tannerella*	· *Rothia*	· *F. nucleatum*
		· *T. forsythia*
		· *Bacteroidetes*
		· *Prevotella species*
		· *Tannerella species*
		
		- The fourth layer:
		· *Spirochaetes* and other bacterial aggregates

Furthermore, dental implants and dental restorative material could be present in the oral cavity, whose chemical and morphological properties enhance the proliferation of bacteria, thus the formation of a new biofilm on dental restorations; in particular, different factors influence the oral biofilm, such as surface roughness of hydrophilicity and material composition [18].

Due to the complexity and variability of oral microorganisms, different methods of qualitative and quantitative analysis should be carried out to study the structure of oral biofilm and its constituents. The oral microbiota composition can be analyzed at various levels, namely at the phylum, genus, species, and genotype levels of the microorganisms constituting the biofilm. Therefore, the techniques for microbiological investigation vary; the techniques of microscopic analysis of the oral biofilm could be divided into two groups: light microscopy and electron microscopy.

Microscopy serves as a fundamental tool in biofilm analysis, offering insights into the structural organization of microbial communities. Different microscopic analysis methods of oral biofilm help assess the microbial community's metabolic activity and viability.

A comprehensive morphological analysis of biofilms necessitates a multi-faceted approach that combines various analytical methods since microscopy techniques offer visual insights, molecular methods provide genetic information, metagenomics elucidates community dynamics, and biochemical analyses unveil the composition of the matrix.

Integrating these diverse methods allows us to understand the physiology of biofilm contributing to advancements in fields ranging from medicine to environmental science [19].

As technology continues to evolve, the synergy of these analytical tools promises even more significant strides in biofilm research.

This review aims to summarize the structure of oral biofilm and illustrate the different microscopy techniques available for biofilm investigation. Indeed, the characterization and the morphological investigation are fundamental from both diagnostic and therapeutic points of view. Thanks to the morphological methods, bacteria could be identified and the efficacy of different treatments to remove oral biofilm could be investigated.

## 2. Microscopy techniques in the study of oral biofilm

The oral biofilm and the microbial community have been studied thanks to the different techniques of light microscopy, such as the optical microscopy associated with Gram stains, the stereomicroscopy, confocal laser scanning microscopy (CLSM), and the methods of electron microscopy, such as transmission electron microscopy (TEM), scanning electron microscopy (SEM), variable pressure scanning electron microscopy (VP-SEM), high resolution-transmission electron microscopy (HR-TEM), scanning and transmission electron microscopy (STEM), scanning electrochemical microscopy (SECM), and the correlative microscopy.

### 2.1. Optical microscopy

#### 2.1.1. What are its advantages and disadvantages in biofilm study?

Light microscopy is an imaging technique that uses visible light to observe and analyze specimens. In the field of oral biofilm investigation, light microscopy offers several advantages: It allows the visualization of the microbial communities in their natural state, providing valuable information about the spatial arrangement of different microorganisms within the biofilm. Another advantage of light microscopy is its ability to reveal the microbial diversity in the biofilm matrix [20]. Disadvantages include the limitation in identifying bacterial ultrastructure components as well as cell counting.

The tools on which light microscopy can rely are stains (Gram and fluorescence probes) and different type of microscopes (light microscope, stereomicroscope, and confocal laser scanning microscope).

#### 2.1.2. Gram stains: uses, advantages, and challenges

Researchers can differentiate and identify various bacterial species by using stains and fluorescent dyes specific to different microorganisms.

Gram staining is a microbiological technique that helps to differentiate bacterial species into two groups based on the characteristics of their cell walls, thus Gram- and Gram+ bacteria, and helps microbiologists to classify bacteria into two major groups, Gram-positive and Gram-negative, based on their ability to retain or lose the crystal violet-iodine complex during decolorization.

Unfortunately, Gram staining is not useful for microorganisms without cell walls (such as mycoplasma) or for microorganisms of reduced dimensions (such as the Chlamydia and Rickettsia species).

The prominent stains used in the Gram-staining procedure are crystal violet, iodine, safranin, and ethanol. Crystal violet is the primary stain used in the Gram-staining process; it stains all cells blue-purple. Iodine (Gram's iodine or Lugol's solution) is a mordant stain applied after the initial staining with crystal violet; it forms a complex with crystal violet within the bacterial cells, enhancing the retention of the stain. Safranin is the counterstain used in Gram staining. After decolorization, gram-negative bacteria are stained pink/red with safranin, while gram-positive bacteria retain the blue-purple color from the crystal violet-iodine complex [21].

Ethanol or ethyl alcohol (a decolorizing agent) is a staining method that differentiates bacteria based on their cell wall structure; in particular, Gram-positive bacteria retain the crystal violet-iodine complex. In contrast, Gram-negative bacteria are decolorized [22].

#### 2.1.3. Stereomicroscopy: uses, advantages, and challenges

Stereomicroscopy provides a three-dimensional view of the sample, allowing researchers to observe the biofilm structure in detail, thus seeing the morphology, thickness, and distribution of microbial communities within the oral biofilm. An advantage of some stereomicroscopes is the live observation, enabling researchers to study dynamic processes within the oral biofilm, such as microbial movement or interactions.

Advanced stereomicroscopes may be combined with features like polarized light, fluorescence, or differential interference contrast, providing additional capabilities for specific analyses [23].

**Figure 2.**
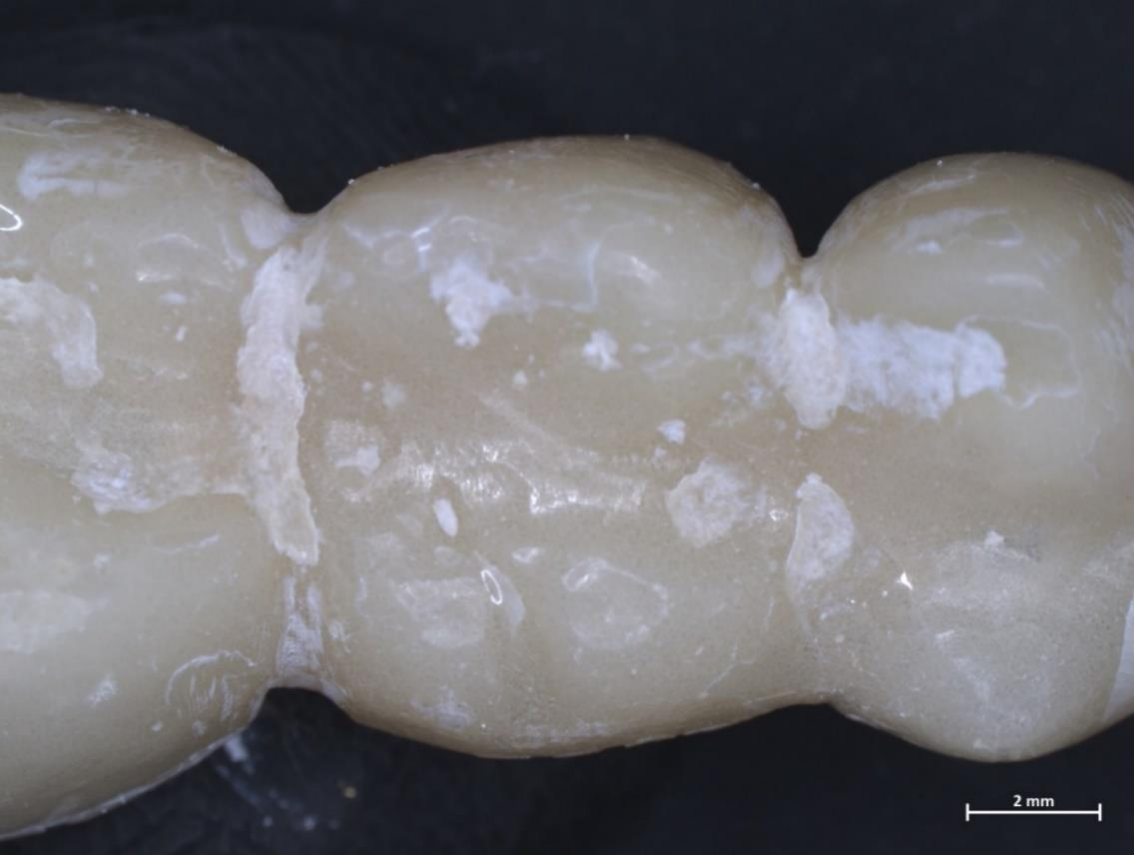
A prosthetic bridge in resin exposed to an oral cavity environment for 7 days. Biofilm traces are difficult to be spotted. Zeiss Axio Zoom V16, 8x magnification.

However, the low magnification and the stereo-view do not allow us to appreciate the fine structures of the microbial community, especially on prosthetic surfaces (Figure 2).

In the study of Hirohata et al., the authors conducted an in vitro study to assess the role of soluble factors produced by *P. gingivalis* on trophoblasts to confirm the correlation between periodontitis and the adverse outcomes of pregnancy. The authors evaluated the morphological changes after cell invasion by using the stereomicroscope. They demonstrated that after 24 hours of culture, the *P. gingivalis* supernatant inhibited the cell invasion without affecting cell viability or inducing apoptosis, but it caused changes in the structural stability of spheroid formations which appeared weak and irregular [24].

#### 2.1.4. Fluorescence in situ hybridization (FISH) and the live/dead method: the roles and challenges of fluorescence probes

Fluorescence in situ hybridization (FISH) has become a fundamental tool, allowing researchers to visualize and directly identify specific microbial populations in their natural habitat.

FISH is a molecular cytogenetic technique that utilizes fluorescently labeled nucleic acid probes to bind complementary target sequences within microbial cells. The method involves the hybridization of these probes under stringent conditions, followed by fluorescence microscopy to visualize the labeled cells. In the context of oral biofilm research, FISH provides a unique opportunity to examine the spatial arrangement and taxonomic distribution of microorganisms within the complex biofilm matrix [25].

FISH enables the spatial mapping of microbial populations in their native spatial context within the oral biofilm and, by targeting specific taxa with fluorescent probes, researchers can observe the distribution and organization of microorganisms on various oral surfaces, providing insights into biofilm structure and architecture. Different probes exist, and many during a microbiological investigation vary according to different needs and the type of microorganisms. In the study of *Bernardi et al*., three different probes were used in order to visualize the microbial community and, in particular, the *Streptococcus* spp. and the *Fusobacterium nucleatum*. The first probe was the EUB 338 probe, which was used to visualize the entire bacterial population within the plaque specimen (Amman 1990; Al-Ahmad et al., 2007), the second one was the FUS 664 probe used to target *F. nucleatum*, and the third one was the STR 405 probe to visualize *Streptococcus* spp. [26].

Furthermore, Karygianni et al. used a method of multiplex fluorescence in situ hybridization (M-FISH) combined with confocal laser scanning microscopy (CLSM) in order to analyze multispecies oral biofilms. The multiplex fluorescence method consisted of using five different probes, which were the oligonucleotide probes EUB 338, E 79, FUS 664, IF 201, and STR 405, applied on *Streptococcus* spp., *Actinomyces naeslundii*, as early colonizers, and *Fusobacterium nucleatum*, *Veillonella* spp., as late colonizers of in situ-formed oral biofilms [27].

The specificity of FISH probes allows for identifying individual microbial species or groups, facilitating a detailed taxonomic analysis of the oral biofilm. In addition, FISH can be tailored to target specific functional groups or metabolic activities within the oral biofilm, allowing researchers to link microbial identity with functional roles, shedding light on the contributions of different taxa to overall biofilm functionality.

Besides, FISH, when combined with image analysis, provides a means to quantify the abundance of specific microbial populations in the oral biofilm [28].

This quantitative information is essential for studying microbial dynamics and the impact of various factors on biofilm composition.

**Figure 3.**
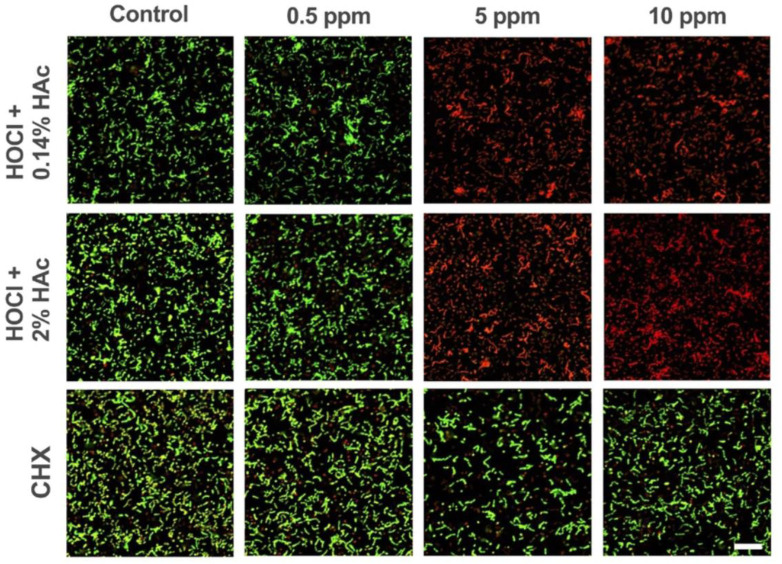
Biofilms treated with concentrations of 0.14% and 2% HOCl, respectively, or CHX, and stained with LIVE/DEAD® BacLight™ viability stain.

As regards live/dead stains, this method provides a real-time assessment of microbial vitality within oral biofilms, offering insights into the proportion of viable and non-viable cells. Live/dead methods are employed to evaluate the efficacy of antimicrobial agents or therapeutic interventions in altering biofilm viability, such as chlorhexidine gluconate (CHX), hypochlorous acid (HOCl) stabilized with acetic acid (HAc). In the study of Aherne et al., the authors investigated the effects of stabilized hypochlorous acid (HOCl) on oral biofilm bacteria. In particular, they exposed *Streptococcus mutans*, *Streptococcus gordonii*, *Actinomyces odontolyticus*, *Veillonella parvula*, *Parvimonas micra*, and *Porphyromonas gingivalis* within a flow-cell HOCl stabilized with 0.14% or 2% HAc, pH 4.6, as well as HOCl or HAc alone. The specimen were analyzed through confocal laser scanning microscopy following LIVE/DEAD® BacLight™ staining (Figure 3). They demonstrated that low concentrations of HOCl (5 ppm), stabilized with 0.14% or 2% HAc, significantly reduced the viability of the oral communities, after 5 minutes, without causing erosion of the HA surfaces [29].

The challenges of these methods are represented by the sample dimensions, the limit of species detection, and the cost of the probes.

Live/dead methods utilize fluorescent dyes that differentially stain live and dead microbial cells based on their membrane integrity. Typically, these dyes include green fluorescent nucleic acid stains for live cells and red fluorescent nucleic acid stains for dead cells. The selective permeability of intact cell membranes allows live cells to retain the green dye, while compromised membranes in dead cells permit the entry of the red dye.

The biofilm is stained with a combination of green and red fluorescent dyes. The choice of dyes depends on the specific assay, with commonly used dyes including SYTO 9 and propidium iodide. In this regard, Tawakoli et al. compared five different live/dead assays, thus fluorescein diacetate (FDA)/propidium iodide (PI), Syto 9/PI (BacLight®), FDA/Sytox red, Calcein acetoxymethyl (AM)/Sytox red, and carboxyfluorescein diacetate (CFDA)/Sytox red, as alternatives to traditional assays which could be instable, such as the ethidium bromide staining method. They demonstrated that BacLight, FDA/Sytox red, Calcein AM/Sytox red, and CFDA/Sytox red were good alternatives to the traditional method based on ethidium bromide staining [30].

#### 2.1.5. Confocal laser microscopy in the analysis of oral biofilm

Confocal laser scanning microscopy (CLSM) provides information on its three-dimensional architecture, microbial composition, and dynamic development. The insights gained through CLSM contribute to our understanding of oral health and hold promise for developing targeted therapeutic strategies to manage biofilm-related diseases [31].

CLSM enables three-dimensional visualization of biofilms, allowing researchers to observe the spatial distribution of different microbial species within the matrix [32].

One of the primary advantages of CLSM lies in its ability to provide high-resolution, three-dimensional images of biofilm structures. In the study of oral biofilm, this capability allows researchers to visualize the arrangement of microbial cells and EPS in unprecedented detail. The non-invasive nature of CLSM permits imaging of live biofilms, capturing the dynamics of biofilm development and response to environmental stimuli.

CLSM facilitates species-specific labeling within oral biofilms through fluorescent probes [33]. This targeted approach enables researchers to distinguish between microbial species and assess their spatial distribution within the biofilm matrix. Fluorescence in situ hybridization (FISH) techniques, when combined with CLSM, allow for the precise identification of specific bacteria within the complex oral microbial community [34].

The real-time imaging capabilities of CLSM make it an invaluable tool for studying the dynamic processes involved in biofilm development. Researchers can observe the initial attachment of bacteria to tooth surfaces, the formation of microcolonies, and the maturation of biofilm architecture over time. This temporal dimension enhances our understanding of the factors influencing biofilm growth and provides crucial insights for developing strategies to manage oral health [35].

Furthermore, CLSM facilitates quantitative analysis of various biofilm parameters, including biomass, thickness, and spatial distribution of microbial populations. Image analysis software allows for quantifying fluorescence signals, providing researchers with data on the abundance and distribution of specific bacteria or biofilm components. This quantitative approach contributes to a more comprehensive understanding of the heterogeneity within oral biofilms [36].

The application of CLSM in studying oral biofilm extends to investigations of biofilm-related diseases, such as dental caries and periodontal diseases. By examining biofilm architecture in disease states, researchers can identify potential targets for therapeutic interventions. Additionally, CLSM is instrumental in evaluating the efficacy of antimicrobial agents or other interventions in disrupting biofilm formation or promoting biofilm removal [37].

Confocal microscopy or flow cytometry can be employed to visualize and quantify the stained cells within the biofilm.

## 3. Electron microscopy

### 3.1. Atomic force microscopy (AFM): uses, advantages, and disadvantages

Atomic force microscopy (AFM) is a high-resolution imaging technique widely used to study various biological samples, including oral biofilms. AFM operates by scanning a sharp tip over the surface of a sample and measuring the interactions between the tip and the sample's surface to create detailed topographical maps, providing a powerful and versatile approach for investigating oral biofilm's nanoscale structure, mechanical properties, and dynamic behavior. Its ability to operate in liquid environments makes it particularly suitable for studying hydrated biological samples like oral biofilms [37]. Its disadvantages are represented by the time employed to get the images and the quality of the image, which can be affected by the geometry of the probe.

AFM produces high-resolution topographical images of the oral biofilm surface revealing details, such as the three-dimensional structure of microbial cells, extracellular polymeric substances (EPS), and biofilm's roughness, thickness, and morphology, and allows us to detect and measure the interactions between microorganisms and substrates and other biofilm components [38].

AFM can be used for force spectroscopy studies, where the tip is used to apply controlled forces to the biofilm surface, allowing researchers to measure the mechanical properties of the biofilm, such as its stiffness or adhesion forces between microbial cells and the substrate [39].

In the study of Tang et al., the mechanism of adhesion of the early colonizers of the oral cavity, *Actynomices* spp., to a substrate at nano newton (nN) range force levels was investigated. The authors measured the interactive forces between the bacterial biofilm formed by fimbriated and non-fimbriated bacteria belonging to the *Actynomices* spp., thus *A. bovis, A. gerencseriae, A. israelii, A. Meyer, A. naeslundii genospecies 1* and *2, A. odontolyticus*, and *A. viscous*, and a silicon nitride tip, through a Nanoscope IIIA atomic force microscope**.** The authors demonstrated that *A. naeslundii genospecies 1*, *2*, and *A. viscosus*, which are fimbriated, develop higher cell-surface interactive forces than fimbriated *Actynimoces* spp. [40].

Furthermore, AFM-based techniques, such as AFM-IR (infrared spectroscopy), can provide chemical information about biofilm components by integrating AFM with infrared spectroscopy, allowing researchers to identify specific molecular structures within the biofilm [41].

### 3.2. Transmission electron microscopy (TEM): uses, advantages, and disadvantages

Transmission electron microscopy (TEM) stands as a fundamental tool in the field of microbiology, allowing the visualization of individual bacterial cells and extracellular polymeric substances (EPS) within oral biofilms at the nanometer scale. This high-resolution imaging capability is crucial for discerning the fine details of biofilm architecture, including the arrangement of bacterial cells, the composition of EPS, and the interactions between microorganisms and matrix components [42],[43].

TEM facilitates a detailed examination of the biofilm matrix, shedding light on the composition and organization of EPS, and allows researchers to identify the different components, such as polysaccharides, proteins, and nucleic acids, contributing to the structural integrity of the biofilm, by visualizing the matrix at the nanoscale. This information is essential to understanding the adhesive and cohesive forces that maintain biofilm stability.

TEM could be applied in the study of antimicrobial treatments, how a molecule may influence the structural organization of the biofilm, and how it acts on microorganisms. The disadvantages of the TEM are represented by the cutting and the protocol used for embedding and the stains.

In the study of Vitkov et al., authors investigated the alterations of the biofilm caused by their exposition to chlorhexidine. The main alterations induced were the disruption of the bacterial membrane integrity and the disintegration of the fimbria, associated with partial matrix disintegration in some cases, but each alteration induced by the chlorhexidine did not induce a whole disruption of the organization of the biofilm, demonstrating that chlorhexidine alone does not have a sufficient efficacy against the oral biofilm [44].

In addition, researchers can explore how bacteria adhere to each other and tooth surfaces, forming microcolonies and establishing the intricate network characteristic of biofilm architecture [45].

Another advantage of TEM in the study of oral biofilm lies in its ability to visualize ultrastructural changes associated with biofilm-related diseases, such as dental caries and periodontal diseases. It allows for the examination of the process on which the alteration of the biofilm matrix and microbial cells is based in disease states, providing critical information for understanding the pathogenesis of these conditions at the nanoscale and investigating new treatments [46]. In the study of Zhao et al., the authors investigated the antimicrobial properties of Mg-Cu alloy grafts in the treatment of bone defects caused by periodontal disease. *P. gingivalis* and *A. actinomycetemcomitans* were cultured in Mg-Cu alloy extracts to evaluate their viability using SEM and TEM. SEM was used to investigate the biofilm changes, while TEM allowed researchers to study its structure. The electron microscopy revealed the complete destruction of the biofilm, resulting in the disruption of cell membranes and the subsequent bacterial apoptosis. In this case, using TEM allowed us to analyze new materials, such as Mg-Cu alloy grafts, against the periodontal bacteria in tissue regeneration [47].

### 3.3. Scanning electron microscopy (SEM): uses, advantages, and disadvantages

Scanning electron microscopy (SEM) is a technique used to study the surface structure of cells and tissues at a high resolution. The SEM technique involves scanning a sample with a focused beam of electrons, and the resulting signals are used to create detailed images of the sample's surface [48].

In the case of oral biofilm, this enables scientists to observe the arrangement, size, and shape of individual bacteria within the biofilm. It provides valuable insights into the composition and organization of microbial communities in the oral cavity, contributing to a better understanding of oral health and diseases. SEM's ability to produce high-resolution images allows for the detailed examination of the surface features of oral biofilms [49].

In the study of Georgiev et al., authors analyzed demineralized enamel specimens infiltrated with triethylene glycol dimethacrylate (TEGDMA) resin and exposed to a biofilm formed by *S. mutans*, *S.oralis*, and *Actinomices oris* in group 1, to the biofilm in group 2, and only resin infiltrated in group 3. A fourth group was used as a control. The authors used SEM and CLSM to study the biofilm and the material's autofluorescence after 24 hours. SEM and CLSM analysis showed reduced biofilm formation on resin-infiltrated specimens (group 1) compared to group 2, while no biofilm was detectable in groups 3 and 4. Thanks to the use of SEM, authors demonstrated that freshly resin-infiltrated enamel surfaces have an effect on biofilm reduction, while monomer leakage was not affected by bacterial presence [50].

**Figure 4.**
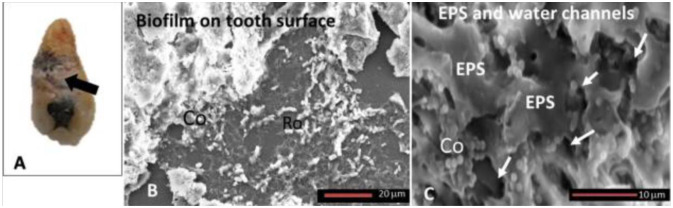
SEM images show the calculus on the tooth surface: (A) Biofilm on the surface with (B) Cocci bacteria (Co) and Rod bacteria (Ro) incorporated into the EPS they produced (C).

The high magnification and depth of field offered by SEM allow researchers to examine the fine details of EPS, including the web-like structures that encapsulate microbial cells. This information is crucial for understanding the biofilm matrix's adhesion, cohesion, and mechanical properties. SEM is employed in detecting, through the auxiliary of other methods like immunohistochemistry, different molecules that could be in the oral biofilm and, in particular, in its structure, such as the EPS, revealing the association between oral pathologies and systemic disease, [51]. In the study of Kanagasingam et al., authors made an SEM analysis in order to detect Amyloid-β (Aβ) in naturally formed oral biofilm: 87 freshly extracted teeth affected by the periodontal disease were selected and collected in two groups, thus, group A of 11 teeth with primary root canal infection and group B with 21 teeth whose endodontic treatment was failed. The biofilm to be analyzed was immune-stained with the anti-Aβ antibody to reveal the eventual presence of Aβ. SEM images helped to characterize the biofilm (Figure 4). Light microscopy associated with immunohistochemistry showed the presence of Aβ in group A, while no specific results were detected in group B [52].

SEM is often coupled with energy-dispersive X-ray spectroscopy (EDS) for elemental analysis, allowing researchers to identify the elemental composition of biofilm components. This integration provides additional information about the chemical composition of microbial cells and the matrix [52]. An important problem during the observation of oral biofilm images with SEM is the dehydration procedure of the specimens, which could induce artifacts such as matrix EPS collapse and misunderstanding in the results. In order to overcome this problem, a recent technique allows us to visualize images with SEM avoiding the process of dehydration but using ionic liquid.

#### 3.3.1. A SEM method combined with an innovative protocol of observation: variable pressure scanning electron microscopy (VP-SEM)

Variable pressure scanning electron microscopy (VP-SEM) is a technique used to overcome the issues in the SEM observations, since it does not require dehydration or drying, but a different fixation with different fixatives represented by aldehydes as glutaraldehyde or paraformaldehyde cross-links proteins and osmium tetroxide (OsO4), which have the advantage of binding to lipids, so that the bacterial and fungal biofilm characterization is more efficient [53].

In the study of Bossù et al. (2020), authors used a different method of characterization of *S. wiggsiase*, which is one of the most representative microorganisms, which composes the early childhood caries (ECC), using SEM combined with a protocol that preserves the biofilm architecture. The protocol developed by the authors, named OsO4-RR-TA-IL, which consists of adopting osmium tetroxide (OsO4), ruthenium red (RR), tannic acid (TA) impregnation, and ionic liquid (IL) drop casting, and allows us to avoid dehydration, drying, and sputter coating. The result of this method is the achievement of high-magnification imaging (from 10000 x to 35000 x) in high-vacuum and high-voltage conditions [54].

### 3.4. Scanning transmission electron microscopy (STEM): uses, advantages, and challenges

Scanning transmission electron microscopy (STEM) is an advanced microscopy technique that combines the principles of TEM and SEM. STEM is beneficial for imaging thin sections of samples with high spatial resolution and is employed in studying various biological specimens, including oral biofilms [55].

STEM provides high spatial resolution imaging, allowing researchers to visualize fine details of the oral biofilm at the nanoscale. It is particularly effective in revealing the internal structures of microbial cells, extracellular polymeric substances (EPS), and other biofilm components. Similar to HR TEM, STEM can be used for three-dimensional imaging by acquiring a series of images from different perspectives. This information can be used to reconstruct three-dimensional representations of the oral biofilm [56].

Hickey et al. used a particular STEM technique, which is bright-field scanning transmission electron microscopy (BF-STEM), combined with tomography to study nanostructures in the biofilm, such as the characteristics of cells that influence the adhesion process and the specific structure that mediate the extracellular communication. The main structures involved are pili, flagella, and membrane tubules. In this kind of investigation, STEM is valuable because it overcomes the limitations of standard TEM, like its defocus issues, so it is possible to reach a high resolution of 4–10 nm in the visualization of the biofilm. STEM images allow us to visualize nanopod structures, deepening the fibrillar formations' shapes and architectures. The advantage of this technique is that it allows for the analysis of the nanorods of bacteria, which contributes to the stability of the biofilm [57].

Another application of STEM is to visualize the antibacterial activity of polymeric nanoparticles on a biofilm with an ionic liquid. Takahashi et al. developed a method to analyze organic polymeric nanoparticles (NPs) in a biofilm formed by *Staphylococcus epidermis*. STEM can show the interaction of drugs with target molecules, which is helpful in studying new aspects of treatments against biofilms [58]. However, few studies are available in the literature, making this a new technique to be explored in the field of biofilm morphological research.

### 3.5. High-resolution transmission electron microscopy (HR-TEM): uses and application

High-resolution transmission electron microscopy (HR-TEM) is an advanced microscopy technique that uses a beam of electrons to create high-resolution images of thin sections of samples. In the study of oral biofilm, HR-TEM can provide detailed insights into the ultrastructure and composition of microbial cells [59].

HR-TEM offers extremely high magnification, allowing researchers to visualize the nanoscale details of microbial cells, EPS, and other structures within the oral biofilm, such as the cell membrane, cell wall, nucleoid, pili, and other cellular organelles. Thanks to its high resolution, HR-TEM is also helpful in studying the antibacterial role of nanoparticles, paving the way for new technologies against oral biofilm. In the study of Azad et al., a new treatment against *E. faecalis*, which is one of the most representative microorganisms in the periapical lesion, based on using bismuth nanoparticles, was analyzed through HR-TEM images showing an actual efficacy against not only *S. mutans*, but also *E. faecalis* whose growth inhibition was demonstrated at a concentration range between 0.625 µg/mL and 20 µg/mL [60].

**Figure 5.**
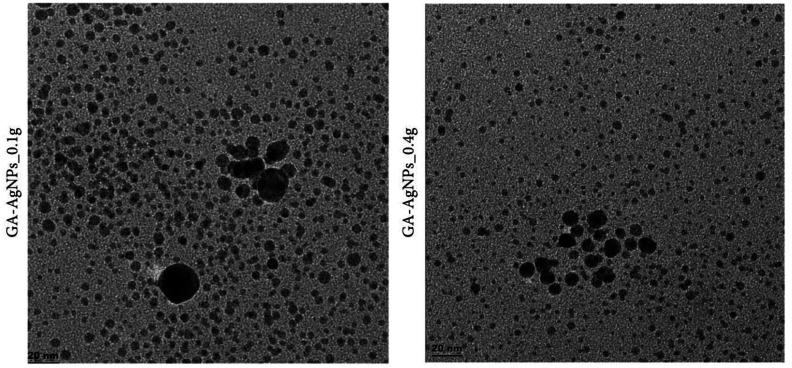
HR-TEM images showing the core size distribution and micrographs of GA-AgNPs.

Another study conducted by Ahmed et al. [61] focused on the antibacterial properties of gum Arabic-silver nanoparticles against *Streptococcus sanguinis* (*S*. *sanguinis*), *Streptococcus mutans* (*S*. *mutans*), *Lactobacillus acidophilus* (*L*. *acidophilus*), and *Candida albicans* (*C*. *albicans*). In this case, HR-TEM was applied to characterize the substrates composed of the gum Arabic-silver nanoparticles, thus its spherical shape with core sizes between 4 and 26 nm. In particular, HR-TEM visualization allowed us to understand that the antimicrobial effect depends on the shape and size of nanoparticles (Figure 5). Moreover, the smaller sizes of gum Arabic-silver nanoparticles showed higher activity than the larger ones [61].

Another example of the application of HR-TEM to study nanoparticles was the research of Jardón-Romero et al., which evaluated the antimicrobial effect of biogenic silver nanoparticles obtained from *Syzygium aromaticum* against *E. coli*, *S. aureus*, *E. faecalis*, *S. mutans*, and *C. albicans*. The HR-TEM images showed that the nanoparticles mentioned above had a crystalline nature and a shape similar to a sphere. Besides, the antibacterial effect had inhibition zones of 2–4 mm in diameter [62].

Some HR-TEM systems are equipped with EDS detectors, which can be used to analyze the elemental composition of the oral biofilm. EDS provides information about the distribution of elements within the biofilm, aiding in identifying specific components [63].

### 3.6. Scanning electrochemical microscopy (SECM): advantages for the biofilm adhesion study

As the chemical microenvironment is crucial for the development of chemical interactions between the oral biofilm and the surface of the niches existing in the oral cavity, an advanced microscopic method that allows researchers to investigate this outstanding aspect of the oral biofilm with a better precision is represented by scanning electrochemical microscopy (SECM). SECM is equipped with a tip represented by a platinum or gold-ultramicroelectrode of about 25 mm [64]. In particular, this method is based on the use of electrochemical sensors, which differs from the traditional ones thanks to their properties of miniaturization, stability, and selectivity. The main electrochemical sensors are Ca^2+^ and hydrogen peroxide, and they are used as probes applied to the scanning electrochemical microscope. The advantage of this method is to obtain the revelation of real-time interaction between the bacterial biofilm and the dental surface [65]. Regarding hydrogen peroxide (H_2_O_2_), its application to the microscopic method is crucial due to its wide presence in many cellular processes as a metabolite. In the study of Joshi et al. [66], the H_2_O_2_ was used as a probe for the electrochemical microscope. In particular, the authors applied Pt nanoparticles on a multi-walled carbon nanotube and conducting ionic liquid matrix in order to obtain high sensitivity for H_2_O_2_ oxidation.

Another type of probe applied to SECM is the dual-tip glucose sensor composed of the glucose oxidase (GOD) enzyme blocked in an ultramicroelectrode (UME). In the study of Jayathilake et al. [67], the above-described glucose microsensor was used in order to obtain the level of local glucose uptake of *S. mutans* biofilms in the presence of sucrose that was metabolised by *S. mutans* to adhere to the tooth surface.

Furthermore, another electrochemical microsensor developed by Joshi et al. in 2017 [68] is a carbon-based pH microsensor. This sensor was applied in order to investigate the microbial metabolic exchange between two prevalent bacteria of the oral biofilm, thus, the commensal *S. gordonii* and pathogenic *S. mutans*. The authors demonstrated that *S. gordonii* was the dominant microorganism producing H_2_O_2_, while *S. mutans* is dominant in pH changes of saliva, resulting in the pH decreasing to 5.0 or less due to the production of lactic acid.

Finally, the combination of different microsensors is possible in scanning electrochemical microscopy, allowing the detection of new real-time events on which the interaction between oral biofilm and tooth surface is based. Developed by Park et al. [69], it combined three sensors—redex, pH, and H_2_O_2_—in order to simultaneously map the pH and the H_2_O_2_ concentration produced by dental plaque on a surface composed of hydroxyapatite.

## 4. Quantitative microscopy: how the image analysis helps in biofilm morphological research

Quantitative microscopy in the study of oral biofilm includes each method combined with the microscopic techniques in order to quantitatively analyze various aspects of oral biofilms, e.g., quantitative microscopy exploits the CLSM and the digital images in order to count the number of nuclei of microorganisms in the specific specimen, and once identified, it allows the counting of the bacterial species. Regarding this type of investigation, another tool used to identify the bacteria and count them is the above-described FISH method.

In the study of Dige et al. [70], in situ biofilm was collected from healthy individuals for 12 hours, divided into two groups in relation to the timing (during the day and during the night). Then, the specimen was treated with the FISH method and bacteria were visualized using confocal laser scanning microscopy. Thanks to the combination of stereological methods and digital images, the authors demonstrated a statistically significant difference between the total number of bacteria and the biovolume in the two 12-hour groups; specifically, the highest accumulation of bacteria was detected during the day. Regarding the use of the FISH method, the authors exploited specific probes for streptococci and *Actinomyces naeslundii* to count them in both daytime and nighttime, showing a higher number of streptococci in biofilms during daytime than nighttime (while there were no differences regarding the amount of *A.Naeslundii*).

Another study conducted by Dige et al. in 2009 [71] showed how the combination of stereological methods with the FISH method was effective in quantifying bacteria. The authors collected samples of intact dental biofilm at 6, 12, 24, 48 hours, which was analyzed through CLSM, while the quantification of bacteria was conducted using 16S ribosomal RNA oligonucleotide probes. The study demonstrated the efficacy of the applied method and showed that the total number of bacteria and streptococci increased over time.

Furthermore, thanks to the development of artificial intelligence (AI) [72], new protocols have been studied in the field of quantitative microscopy. In a recent study conducted by Ding et al., an AI method combined with SEM was applied in order to quantify the initial bacterial adhesion on different dental materials. In particular, authors measured the amount of *Porphyromonas gingivalis* and *Fusobacterium nucleatum* on dental zirconia surfaces using SEM images at 1, 7, and 24 hours to which Fiji software, as the AI method, was applied. The same software was used on SEM images of *Streptococcus mutans* cultured on a PMMA nanostructured surface at 1, 24, 72, and 168 hours. It was then compared with the traditional quantitative microscopy applied to the live/dead CLSM method.

The results showed an increasing amount of bacteria over time, while both the AI method and CLSM were comparable, paving the way for a reduction of time, cost, and labor thanks to the use of AI software combined with SEM images.

## 5. Correlative microscopy

Correlative microscopy involves the synergistic application of multiple imaging techniques to study biological specimens [73].

In oral biofilm research, this approach combines the strengths of different microscopy modalities, such as light, electron, and fluorescence. By employing techniques such as confocal laser scanning microscopy in tandem with scanning electron microscopy or light microscopy combined with high-resolution electron microscopy, researchers can achieve a seamless transition from macro- to micro-scale observations [74].

The combination of fluorescence and electron microscopy in correlative studies enables researchers to unravel the microbial diversity within oral biofilm. Fluorescence microscopy, capable of selectively labeling specific microbial groups, can identify critical bacterial species. Subsequently, electron microscopy provides ultrastructural details, allowing for a more in-depth analysis of microbial interactions and spatial organization within the biofilm matrix [75].

As demonstrated by Daddi Oubekka et al., correlative time-resolved fluorescence microscopy is a method to describe the diffusion of antibiotics in biofilm. Many factors related to the microorganism and the architecture of the biofilm can be obstacles to the diffusion of antibiotics, so the advantage of this technique is to obtain images in an accurate spatiotemporal resolution, overcoming the limitations of the time-lapse confocal imaging microscopy, in order to assess the characteristics of the biofilm and how it reacts to antibiotics. The set of techniques used in this study was composed of fluorescence recovery after photobleaching, fluorescence correlation spectroscopy, and fluorescence lifetime imaging. The correlative analysis was conducted on a biofilm formed by two *S. aureus* to assess the reaction to the diffusion of vancomycin in the biofilm. The authors showed that the matrix that composes the biofilm did not obstruct the diffusion of vancomycin, which could penetrate through all cells. However, biofilm components could be obstacles for the bioavailability of vancomycin, which is the real reason why many antibiotics have a low efficacy when their target is the biofilm [76].

## 6. Conclusions

The complexity of oral biofilm requires different methods of investigation, both for understanding its origins and how to control it. The above-described microscopy techniques have been developed to exploit the advantages of each one in the study of the different microscopical levels of biofilm. The established microscopy methods (light, fluorescence, and electron) have been widely used, with the development of specific protocols to allow for a high-quality morphological characterization and visualization. On the other hand, the most recent microscopy methods, such as STEM, HR-TEM, and correlative microscopy, have yet to be fully exploited for studying oral biofilm and the efficacy of nanomolecules used for microbicidal purposes. Further protocols and studies on applying advanced microscopic techniques and image analysis are strongly necessary to obtain fine details on the microbiological, pathological, and therapeutic aspects of oral biofilm.

## Use of AI tools declaration

The authors declare they have not used Artificial Intelligence (AI) tools in the creation of this article.
